# Circulating tumor DNA predicts survival in patients with resected high-risk stage II/III melanoma

**DOI:** 10.1093/annonc/mdx717

**Published:** 2017-11-03

**Authors:** R J Lee, G Gremel, A Marshall, K A Myers, N Fisher, J A Dunn, N Dhomen, P G Corrie, M R Middleton, P Lorigan, R Marais

**Affiliations:** 1 Molecular Oncology Group, Cancer Research UK Manchester Institute, University of Manchester, Manchester, UK; 2 Warwick Clinical Trials Unit, University of Warwick, Coventry, UK; 3 Oxford Experimental Cancer Medicine Centre, University of Oxford, Oxford, UK; 4 Cambridge University Hospitals NHS Foundation Trust, Cambridge, UK; 5 Faculty of Biology, Medicine and Health, The University of Manchester, Manchester, UK; 6 Department of Medical Oncology, Christie NHS Foundation Trust, Manchester, UK

**Keywords:** circulating tumor DNA, melanoma, adjuvant, prognosis

## Abstract

**Background:**

Patients with high-risk stage II/III resected melanoma commonly develop distant metastases. At present, we cannot differentiate between patients who will recur or those who are cured by surgery. We investigated if circulating tumor DNA (ctDNA) can predict relapse and survival in patients with resected melanoma.

**Patients and methods:**

We carried out droplet digital polymerase chain reaction to detect *BRAF* and *NRAS* mutations in plasma taken after surgery from 161 stage II/III high-risk melanoma patients enrolled in the AVAST-M adjuvant trial.

**Results:**

Mutant *BRAF* or *NRAS* ctDNA was detected (≥1 copy of mutant ctDNA) in 15/132 (11%) *BRAF* mutant patient samples and 4/29 (14%) *NRAS* mutant patient samples. Patients with detectable ctDNA had a decreased disease-free interval [DFI; hazard ratio (HR) 3.12; 95% confidence interval (CI) 1.79–5.47; *P *<* *0.0001] and distant metastasis-free interval (DMFI; HR 3.22; 95% CI 1.80–5.79; *P *<* *0.0001) versus those with undetectable ctDNA. Detectable ctDNA remained a significant predictor after adjustment for performance status and disease stage (DFI: HR 3.26, 95% CI 1.83–5.83, *P *<* *0.0001; DMFI: HR 3.45, 95% CI 1.88–6.34, *P *<* *0.0001). Five-year overall survival rate for patients with detectable ctDNA was 33% (95% CI 14%–55%) versus 65% (95% CI 56%–72%) for those with undetectable ctDNA. Overall survival was significantly worse for patients with detectable ctDNA (HR 2.63; 95% CI 1.40–4.96); *P *=* *0.003) and remained significant after adjustment for performance status (HR 2.50, 95% CI 1.32–4.74, *P *=* *0.005).

**Conclusion:**

ctDNA predicts for relapse and survival in high-risk resected melanoma and could aid selection of patients for adjuvant therapy.

**Clinical trial number:**

ISRCTN 81261306

Key Message

Detectable circulating tumor DNA postsurgery for high-risk stage II/III melanoma is predictive of relapse and survival, independent of standard prognostic indices. Future adjuvant trials should analyze for treatment effect in this poor prognostic group of patients.

## Introduction

Many patients with loco-regional melanoma will subsequently develop distant metastases; however, current predictors of relapse are relatively crude. Currently, American Joint Committee on Cancer (AJCC) staging of loco-regional melanoma at the time of surgery is used to identify different risk groups and can inform decisions on intensity of follow-up, potential adjuvant therapy and inclusion in clinical trials. However, there are significant limitations because staging is based on a single snapshot of anatomical or histological features and then used as a surrogate for the biological behavior of the tumor over time. Patients are divided into broad prognostic groups without sufficient information to accurately predict the likely outcome on an individual patient basis. This is particularly relevant in light of the recent approval of adjuvant ipilimumab in melanoma. Ipilimumab (10 mg/kg) was associated with 11% improvement in overall survival (OS) at 5 years, from 54.4% to 65.4%, but at the expense of grade 3/4 toxicity in >54% of patients, including five (1.1%) treatment-related deaths [1]. This is in a patient population that is potentially cured by surgery alone.

It is, therefore, important to develop tools that can accurately identify patients who are at highest risk of progression to stage IV disease. Circulating tumor DNA (ctDNA, the tumor-derived fraction of circulating free DNA or cfDNA) is emerging as a useful measure of tumor burden and prognostic marker in stage IV melanoma [2, 3]. This study aims to determine whether having detectable ctDNA within 12 weeks of surgery carried out with curative intent for high-risk stage II/III disease was associated with worse survival in a subgroup of patients whose tumors were known to have either a *BRAF* or *NRAS* mutation.

## Methods

### Study design

Samples were collected as part of the AVAST-M trial (ISRCTN 81261306), which compared bevacizumab versus placebo in 1343 patients with resected high-risk stage II/III melanoma [4]. This study reported a difference in disease-free interval (DFI) between trial arms [hazard ratio (HR) 0.83; 95% confidence interval (CI) 0.70–0.98, *P *=* *0.03] but no impact on distant metastasis-free interval (DMFI) or OS [4]. Patients with a confirmed *BRAF* or *NRAS* mutation were randomly selected from both arms of AVAST-M. Work was carried out in accordance with the Declaration of Helsinki (supplementary Methods, available at *Annals of Oncology* online).

### Sample size

In this retrospective analysis, 150 patients were determined to provide 80% power to detect a HR of at least 3.5 between patients with undetectable and detectable ctDNA for DFI with a 5% significance level, assuming a 10% marker prevalence and an event rate of 40%.

### Analysis of ctDNA

Mutational status was determined using several different methods including pyro-sequencing of formalin fixed paraffin embedded tissue from the resected primary lesion or involved lymph node, or both where available. Discordant results were repeated in triplicate to provide a consensus result. Baseline plasma samples were taken within 12 weeks (median 8.3 weeks from surgery to blood draw; range 2.4–12 weeks) of surgical clearance for stage IIB, IIC or III melanoma. cfDNA was isolated from up to 2 ml of plasma (individual patient details in supplementary Table S1, available at *Annals of Oncology* online) using QIAamp Circulating Nucleic Acid kits according to the manufacturer’s instructions (Qiagen, Hilden, Germany) and droplet digital PCR (ddPCR) carried out using a QX200 ddPCR system (Bio-Rad, details in supplementary Methods, available at *Annals of Oncology* online). ctDNA was defined as detectable if there was  ≥1 copy of mutant DNA detected.

### Statistical analysis

The baseline ctDNA result (undetectable/detectable) was compared against patient and tumor characteristics [age, gender, AJCC stage, nodal classification, primary melanoma Breslow and ulceration, as well as Eastern Cooperative Oncology Group performance status (ECOG PS)] collected at the time of trial entry using Wilcoxon rank sum test for continuous factors and a chi-square test or Fisher’s exact test with small number for categorical factors. A stepwise logistic regression model was used to identify the independent factors for predicting detectable ctDNA, with a *P*-value of 0.05 for inclusion and exclusion.

DFI, DMFI and OS were calculated from the date of randomization to the trial until date of first recurrence, date of distant metastases and date of death, respectively. The Kaplan–Meier method was used to construct survival curves for differences between DFI, DMFI and OS in patients with detectable ctDNA levels versus undetectable levels and compared using a Cox proportional hazards model to obtain HRs and 95% CIs. Baseline ctDNA (detectable or undetectable) and other factors associated with prognosis (Breslow, ulceration, stage, nodal classification and ECOG PS) were analyzed using univariate and multivariate Cox proportional hazards regression models for DFI, DMFI and OS. Details regarding internal validation of ctDNA and performance modeling can be found in supplementary Methods, available at *Annals of Oncology* online. All analyses were carried out using the SAS statistical package (version 9.4).

## Results

### Patient demographics and detection of baseline ctDNA

To evaluate the potential for ctDNA to identify melanoma patients at high risk of relapse following surgery with curative intent, we analyzed ctDNA in the plasma from 161 patients carrying either a *BRAF* or *NRAS* mutation in their baseline resected tumor (Table 1 and supplementary Tables S1 and S2, available at *Annals of Oncology* online). Patient demographics are presented in Table 1. Within the cohort, 132 tumors had a p.V600E *BRAF* mutation and 29 tumors had a p.Q61L/K *NRAS* mutation. CtDNA was detected in 19 (12%) of the plasma samples (10 from the treatment arm and 9 from the observation arm). Of the 19 positive plasma samples, 15 had a p.V600E *BRAF* mutation and 4 had a p.Q61L/K *NRAS* mutation. The Poisson-corrected ctDNA levels ranged from 1.4 to 1608 copies, with a median of 2.8 (supplementary Tables S1 and S2, available at *Annals of Oncology* online).

**Table 1.** Demographics of patients with detectable or undetectable ctDNA

Characteristic	Total	Undetectable ctDNA	Detectable ctDNA
	*N* (%)	*N* (%)	*N* (%)
Age in years, median (range)	52 (19–87)	52 (19–79)	59 (22–87)
*P* value	0.29		
Gender			
Male	77 (48)	70 (49)	7 (37)
Female	84 (52)	72 (51)	12 (63)
*P* value	0.31		
Breslow of primary tumor			
≤2.0 mm	61 (38)	53 (37)	8 (42)
>2–4.0 mm	49 (30)	43 (30)	6 (32)
>4.0 mm	42 (26)	38 (27)	4 (21)
Unknown	9 (6)	8 (6)	1 (5)
*P* value	0.96		
Ulceration of primary tumor			
Present	63 (39)	57 (40)	6 (32)
Absent	77 (48)	69 (49)	8 (42)
Unknown	21 (13)	16 (11)	5 (26)
*P* value	0.19		
Disease stage			
II	36 (22)	33 (23)	3 (16)
IIIA	29 (18)	27 (19)	2 (11)
IIIB	59 (37)	51 (36)	8 (42)
IIIC	37 (23)	31 (22)	6 (32)
*P* value	0.61		
Nodal classification			
II (No or N/A)	36 (22)	33 (23)	3 (16)
III (N1a and N2a)	41 (26)	36 (25)	5 (26)
III (other N)	84 (52)	73 (52)	11 (58)
*P* value	0.81		
ECOG PS			
0	138 (86)	125 (89)	13 (68)
1	22 (14)	16 (11)	6 (32)
*P* value	0.03		
Mutation status			
BRAF V600E	132 (82)	117 (82)	15 (79)
NRAS Q61K/L	29 (18)	25 (18)	4 (21)
*P* value	0.75		
Trial arm			
Bevacizumab	81 (50)	71 (50)	10 (53)
Observation	80 (50)	71 (50)	9 (47)
*P* value	0.83		
Total	161 (100)	142 (88)	19 (12)

ctDNA, circulating tumor DNA; ECOG PS, Eastern Cooperative Oncology Group performance status; N, number.

*P* values were obtained using the Wilcoxon rank sum test for continuous factors and a chi-square test or Fisher's exact test with small number for categorical factors.

In univariate analyses of known prognostic factors, only PS was identified as significantly associated with detectable ctDNA (*P *=* *0.03) (Table 1). This was confirmed using a multivariate logistic regression. There was a significantly increased chance of having positive ctDNA in patients with PS 1 compared with 0 (odds ratio = 3.61; 95% CI 1.20–10.82, *P *=* *0.02).

### Patient outcomes

At a median of 5 years, 21% (95% CI 7–41%) of patients with detectable ctDNA were alive and recurrence free compared with 49% (95% CI 40%–57%) for those with undetectable ctDNA. Of the four patients who did not recur, one patient had stage II disease, one patient stage IIIA and two patients had stage IIIB disease, three patients were on the treatment arm and one patient on the observation arm. All nine patients with >3 mutant copies have recurred (one patient had regional lymph nodes metastases; two patients recurred distantly only and six patients had both loco-regional recurrence and distant metastases). Fifty-two percent (74/142) of patients with undetectable ctDNA have recurred (patterns of the relapses/outcomes are presented in supplementary Tables S3 and S4, available at *Annals of Oncology* online). Twelve (63%) of the 19 patients with detectable ctDNA are known to have died compared with 49 (35%) of the 142 patients without detectable ctDNA.

### Prognostic significance of detectable ctDNA

**Figure 1.**
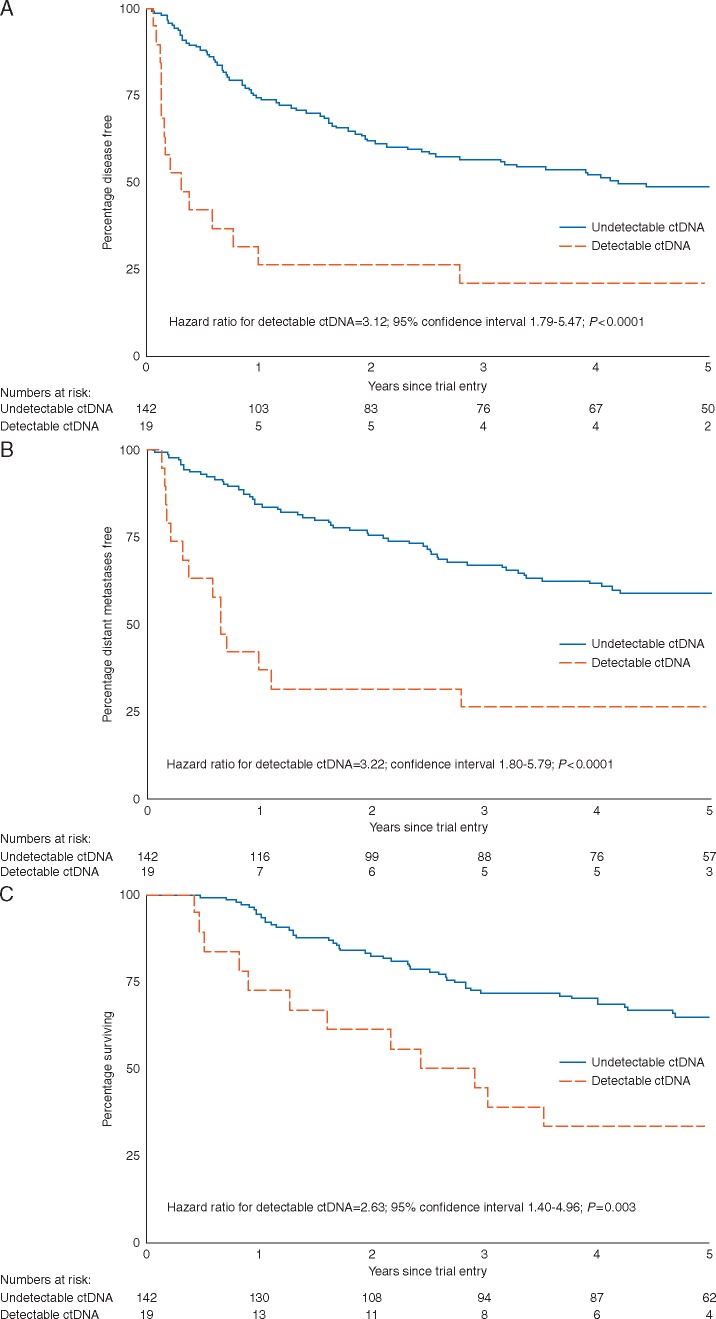
Kaplan-Meier curves for (A) Disease-free interval (DFI). Median DFI was 0.3 years (95% CI 0.1–1.0) in patients with detectable ctDNA compared with 4.2 years (2.5–limit not reached) in those with undetectable ctDNA. (B) Distant metastasis-free interval (DMFI). Median DMFI was 0.6 years (95% CI 0.2–2.8) with detectable ctDNA compared with the median not reached (95% CI 5.0–limit not reached) for those with undetectable ctDNA. (C) Overall survival (OS). Median OS was 2.9 years (95% CI 0.9–limit not reached) with detectable ctDNA compared with the median not reached for those with undetectable ctDNA (95% CI 6.0–limit not reached).

Median DFI was 0.3 years (95% CI 0.1–1.0) in patients with detectable ctDNA compared with 4.2 years (95% CI 2.5–limit not reached) in those where ctDNA was not detected (Figure 1A). There was no significant interaction between trial arm and the ctDNA in predicting DFI (*P *=* *0.60) (supplementary Analysis S5, available at *Annals of Oncology* online). Patients with detectable ctDNA had significantly increased risk of recurrence compared with those with undetectable ctDNA [HR for detectable ctDNA 3.12; 95% CI 1.79–5.47; *P *<* *0.0001; prognostic separation D statistics (PSDS) = 0.97; standard error (SE) = 0.24; Table 2]. Bootstrapping provided internal validation of ctDNA, with ctDNA being a significant predictor of DFI in 92% of the bootstrapped samples (PSDS = 0.99, SE = 0.24). At 1 year, 26% (95% CI 10%–47%) of the patients with detectable ctDNA were disease free compared with 74% (95% CI 66%–81%) for patients with undetectable ctDNA (Table 2). Sensitivity for predicting relapse was 18% and specificity 95%, with a positive predictive value of 79% and negative predictive value of 51%.

**Table 2.** Univariate Cox proportional hazards regression analysis for prediction of disease-free interval (DFI), distant metastasis-free interval (DMFI) and overall survival (OS)

Parameter	DFI				DMFI				OS			
	% DF	% DF at 1 year (95% CI)	Univariate analysis		% DMF	% DMF at 1 year (95% CI)	Univariate analysis		% alive	% alive at 1 year (95% CI)	Univariate analysis	
			*P*	HR (95% CI)			*P*	HR (95% CI)			*P*	HR (95% CI)
ctDNA			<0.0001				<0.0001				0.003	
Undetectable	48	74 (66–81)		1.00	58	84 (77–89)		1.00	65	94 (89–97)		1.00
Detectable	21	26 (10–47)		3.12 (1.79–5.47)	26	37 (17–57)		3.22 (1.80–5.79)	37	72 (46–88)		2.63 (1.40–4.96)
Breslow			0.51				0.67				0.42	
≤2.0 mm	48	65 (52–76)		1.00	51	75 (62–84)		1.00	57	90 (79–95)		1.00
>2–4.0 mm	49	69 (54–80)		0.94 (0.56–1.58)	59	79 (65–88)		0.79 (0.45–1.40)	67	91 (79–97)		0.76 (0.41–1.42)
>4.0 mm	33	69 (53–81)		1.27 (0.77–2.11)	50	81 (65–90)		0.96 (0.55–1.68)	57	95 (82–99)		0.98 (0.54–1.79)
Unknown	56	89 (43–98)		ND	67	89 (43–98)		ND	89	89 (43–98)		ND
Ulceration			0.94				0.93				0.57	
Present	43	71 (58–81)		1.00	54	77 (65–86)		1.00	59	92 (81–96)		1.00
Absent	45	67 (56–77)		0.96 (0.62–1.50)	53	82 (71–89)		0.97 (0.60–1.58)	62	95 (86–98)		0.86 (0.50–1.45)
Unknown	48	67 (43–83)		ND	57	71 (47–86)		ND	71	81 (57–92)		ND
Disease stage			0.03				0.03				0.14	
II	56	86 (69–94)		0.47 (0.25–0.88)	64	91 (76–97)		0.45 (0.23–0.89)	67	97 (81–100)		0.60 (0.28–1.25)
IIIA	59	79 (60–90)		0.42 (0.21–0.84)	69	93 (75–98)		0.37 (0.17–0.80)	79	96 (77–99)		0.37 (0.15–0.94)
IIIB	41	62 (49–73)		0.75 (0.45–1.25)	51	74 (61–84)		0.67 (0.39–1.16)	56	91 (81–96)		0.86 (0.47–1.58)
IIIC	30	54 (37–68)		1.00	38	62 (45–76)		1.00	54	83 (67–92)		1.00
Nodal classification			0.06				0.12				0.38	
II (No or N/A)	56	86 (69–94)		0.56 (0.32–0.99)	64	91 (76–97)		0.58 (0.32–1.08)	67	97 (81–100)		0.69 (0.36–1.33)
III (N1a and N2a)	51	73 (57–84)		0.64 (0.38–1.07)	61	83 (67–91)		0.64 (0.36–1.14)	68	93 (79–98)		0.70 (0.37–1.32)
III (other N)	37	59 (48–69)		1.00	46	71 (60–80)		1.00	57	89 (80–94)		1.00
ECOG PS			0.02				0.01				0.01	
0	48	73 (65–80)		0.51 (0.30–0.88)	57	83 (76–88)		0.46 (0.26–0.83)	66	96 (91–98)		0.43 (0.23–0.80)
1	27	45 (24–64)		1.00	36	54 (32–72)		1.00	41	68 (44–83)		1.00

Median DMFI was 0.6 years (95% CI 0.2–2.8) with detectable ctDNA, but was not reached even with 5-year follow-up (95% CI 5.0–limit not reached) for those with undetectable ctDNA (Figure 1B). Patients with detectable ctDNA had a significantly increased risk of distant metastatic recurrence compared with those with undetectable ctDNA (Table 2, HR 3.22; 95% CI 1.80–5.79; *P *<* *0.0001, PSDS = 0.99, SE = 0.25). Bootstrapping confirmed ctDNA as a significant predictor of DMFI in 92% of samples (PSDS =1.03, SE = 0.26). At 1 year, 37% (95% CI 17%–57%) of the patients with detectable ctDNA were free of distant metastases compared with 84% (95% CI 77%–89%) for patients with undetectable ctDNA (Table 2). Sensitivity for predicting distant relapse was 20% and specificity 95% with a positive predictive value of 74% and negative predictive value of 61%.

OS was significantly worse for the 19 patients that had detectable ctDNA compared with the 142 with undetectable ctDNA (Table 2, HR 2.63; 95% CI 1.40–4.96; *P *=* *0.003, PSDS = 0.82, SE = 0.27). Bootstrapping confirmed ctDNA as a significant predictor of OS in 81% of samples (PSDS = 0.83, SE = 0.26). Median OS was 2.9 years (95% CI 0.9–limit not reached) with detectable ctDNA compared with median not reached with 5-year follow-up for those with undetectable ctDNA (95% CI 6.0–limit not reached, Figure 1C). At 1 year, 72% (95% CI 46%–88%) of patients with detectable ctDNA were alive compared with 94% (95% CI 89%–97%) for patients with undetectable ctDNA (Table 2). At 5 years, 33% (95% CI 14%–55%) of patients with detectable ctDNA were alive compared with 65% (95% CI 56%–72%) for those with undetectable ctDNA. Of note, only 12 patients (none in the ctDNA detectable group) received targeted or immune therapy on relapse due to limited availability of these treatments at the time of the study (supplementary Table S3, available at *Annals of Oncology* online). Results were similar within the *BRAF* and *NRAS* mutant subgroups for all above perimeters (data not shown).

### Association of prognostic factors and ctDNA on outcome

In univariate analysis ctDNA (*P *<* *0.0001) was significantly more predictive of DFI than either PS (*P *=* *0.02) or disease stage (*P *=* *0.03) (Table 2), and none of the other known prognostic factors (Breslow, ulceration, nodal classification) were significant. Similarly, ctDNA (*P* ≤ 0.0001) was significantly more predictive of DMFI than PS (*P *=* *0.01) or disease stage (*P *=* *0.03) (Table 2). Critically, in a multivariate Cox proportional hazards regression model, ctDNA remained a significant predictor for DFI (HR 3.26, 95% CI 1.83–5.83, *P *<* *0.0001) and DMFI (HR 3.45, 95% CI 1.88–6.34, *P *<* *0.0001) after adjustment for PS and disease stage (Table 3). For OS, in univariate analyses, ctDNA (*P *=* *0.003) was significantly more predictive than PS (*P *=* *0.01), and disease stage was not predictive, nor were other factors associated with AJCC staging (Table 2). In multivariate analysis, ctDNA remained a significant predictor of OS after adjustment for PS (HR 2.50, 95% CI 1.32–4.74, *P *=* *0.005, Table 3). Finally, to compare the performance of ctDNA in addition to standard prognostic factors, we modeled the prognostic ability of variables associated with AJCC staging (stage, nodal classification, ulceration, Breslow) and then adjusted for ctDNA (Table 4). When adjusted for ctDNA, all indices (PSDS, Nagelkerke’s *R*^2^, Calibration shrinkage measure) showed significantly improved prognostic value for DFI, DMFI and OS (Table 4).

**Table 3.** Multivariate Cox proportional hazards regression analysis for prediction of DFI, DMFI and OS

Parameter	DFI		DMFI		OS	
	*P*	HR (95% CI)	*P*	HR (95% CI)	*P*	HR (95% CI)
ctDNA	<0.0001		<0.0001		0.005	
Undetectable		1.00		1.00		1.00
Detectable		3.26 (1.83–5.83)		3.45 (1.88–6.34)		2.50 (1.32–4.74)
ECOG	0.02		0.01		0.02	
0		0.52 (0.30–0.89)		0.46 (0.25–0.82)		0.47 (0.25–0.87)
1		1.00		1.00		1.00
Disease stage	0.02		0.02			
II		0.46 (0.25–0.87)		0.45 (0.23–0.89)		
IIIa		0.38 (0.19–0.76)		0.34 (0.16–0.74)		
IIIb		0.66 (0.39–1.12)		0.59 (0.34–1.04)		
IIIc		1.00		1.00

**Table 4.** Model performance measures for the staging variables associated with AJCC classification (stage, Nodal classification, ulceration and Breslow) and the model adjusted for ctDNA

Model Outcome Measure	AJCC staging variables			Adjusted for ctDNA		
	DFI	OS	DMFI	DFI	OS	DMFI
Prognostic separation measure D statistic	0.63 (SE = 0.17)	0.70 (SE = 0.21)	0.53 (SE = 0.18)	0.96 (SE = 0.20)	0.98 (SE = 0.23)	1.01 (SE = 0.22)
Predictive ability measure Nagelkerke’s *R*^2^	0.093	0.085	0.077	0.17	0.13	0.15
Calibration shrinkage measure	0.43	0.36	0.29	0.65	0.53	0.63

SE, standard error.

## Discussion

In the evolving paradigm of effective adjuvant therapy in melanoma, it is essential to develop biomarkers identifying patients at high risk of relapse. Currently, features of the primary tumor such as ulceration, Breslow and number of mitoses in addition to nodal classification and disease stage are standard measures to predict melanoma progression [5]. Furthermore, gene expression profiling has identified subsets that are associated with a poor outcome in stages I–III melanoma; however, patient numbers in these studies were small and have yet to be confirmed in larger cohorts [6, 7].

In this study, we showed that detecting ctDNA in plasma taken within 12 weeks of curative intent surgery is highly predictive of relapse in patients with stage II/III melanoma. The majority of patients with detectable ctDNA relapsed within 1 year of surgery suggesting that ctDNA in the plasma can reveal occult metastatic disease that is not evident on radiological imaging. Notably, we were able to identify melanoma patients at high risk of both distant metastatic relapse and local recurrence, which is consistent with studies showing that ctDNA can signal micrometastatic disease after neoadjuvant chemotherapy postsurgical resection in breast cancer, and following surgery for stage II colorectal cancer [8, 9].

Critically, our findings were independent of standard staging indices, demonstrating the value of this approach in melanoma. It is reported that PS is an independent predictor for DFI, DMFI and OS in the AVAST-M study population [4]. Although the reasons for this intriguing observation are not known, even when adjusted for PS in multivariate analysis, ctDNA was significant in predicting DFI, DMFI and OS. Moreover, in this cohort, AJCC variables were a poor predictor of relapse, but when we created a model in which the standard AJCC variables were adjusted for ctDNA, the performance improved significantly. The test was specific, but not sensitive and therefore should be seen as an adjunct to current AJCC staging when discussing risk of relapse and adjuvant options for the individual patient.

The patients evaluated in this study were treated in an era where access to immune and targeted therapies was limited. It will be important to determine whether outcome can be improved in this extremely poor prognostic subgroup using targeted and immune therapies in future clinical trials. In stage IV melanoma, baseline ctDNA levels in patients treated with both targeted and immune therapies have been shown to correlate with inferior survival and disease burden [2, 3]. Taken together, these data show that ctDNA levels during the course of disease reflect disease biology and are associated with patient outcome.

A minimally invasive, blood test based on ddPCR, which is simple, relatively inexpensive and could be carried out within 5 days, is particularly advantageous in the clinical setting especially when compared with next generation sequencing of tumor material, which is time-consuming, costly and requires specialist skills to perform and analyze. ddPCR is extremely sensitive and can reach detection sensitivities of approximately 0.01% [10] and we were able to demonstrate significance with only 2 ml of patient plasma. Moreover, our proof-of-principle study focused on the driver mutations *BRAF* and *NRAS*, which account for up to 70% of melanomas, and are well suited to this purpose because of the low likelihood of clonal diversity with trunk mutations such as these in melanoma. Furthermore, driver mutations usually have the highest variant allele frequency, which improves the sensitivity of the test. To analyze ctDNA in patients without *BRAF* or *NRAS* mutations, next generation sequencing of the primary tumor/lymphadenectomy can be used to identify the driver/trunk mutations in individual patients and create bespoke panels.

Critically, the strength of this retrospective study is that it allows sufficient follow-up to identify the patients who relapsed, using small amounts of sample. Clearly, prospective studies in the adjuvant setting will be needed to validate these findings and examine treatment effect of new agents. Based on the findings of others [8] and the low sensitivity seen in this assay, it is unlikely that a single time-point following surgery will identify all patients who are going to relapse, but we propose that longitudinal sampling will resolve this issue and improve the sensitivity. Longitudinal sampling has identified treatment relapse before radiological imaging in stage IV melanoma providing a rationale for such an approach [3]. Furthermore, longitudinal sampling will reduce the likelihood of false-positive results. For one of the patients with detectable ctDNA within 12 weeks who did not relapse within the follow-up period, subsequent analysis was found to be negative for ctDNA at 3, 6, 12, 18, 24, 36, 48 and 60 months, and therefore confirmatory testing should be mandatory for this assay to be part of clinical decision-making.

The ability to predict progression to stage IV disease is extremely important in light of recent findings that immune checkpoint inhibition improves OS in stage III melanoma [1]. Detection of ctDNA allows identification of a subgroup of patients at high risk of early relapse and inferior survival, allowing stratification of patients to adjuvant regimens associated with higher toxicity but greater potential for efficacy [11]. Taken at a single time-point following surgery, it can add to AJCC staging in informing individual prognosis and therefore discussion regarding risks and benefits of adjuvant therapy, while longitudinal sampling will likely improve the ability to detect disease progression before radiological imaging. We advocate that our findings be confirmed in clinical trials investigating treatment responses in this population in order to evaluate whether it is also a predictive biomarker for response to immune or targeted therapy.

## Supplementary Material

Supplementary Analysis

Supplementary Methods

Supplementary Tables
