# Supplementary material for: Circulating tumor DNA predicts survival in patients with resected high-risk stage II/III melanoma
Source: Ann Oncol. 2017 Nov 3;29(2):490–6. doi: 10.1093/annonc/mdx717 (PMC5834029; doi:10.1093/annonc/mdx717)
Supplement: Supplementary Analysis [file supplementary_analysis_s5_mdx717.docx]

**Supplementary analysis (S5).**

In the AVAST-M trial, within this subgroup of 161 patients (total trial population 1343), trial arm had a borderline effect on DFI in univariate analysis (HR=0.68; 95% CI 0.45-1.03, p=0.07). After adjusting for trial arm, ctDNA is still a highly significant predictor of DFI (p<0.0001). Also after adjusting for PS, disease stage and trial arm, ctDNA remained a significant predictor for DFI (HR=3.31, 95% CI 1.84-5.94, P<0.0001).
