# Supplementary material for: Circulating tumor DNA predicts survival in patients with resected high-risk stage II/III melanoma
Source: Ann Oncol. 2017 Nov 3;29(2):490–6. doi: 10.1093/annonc/mdx717 (PMC5834029; doi:10.1093/annonc/mdx717)
Supplement: Supplementary Methods [file supplementary_methods_mdx717.docx]

**Supplementary methods**

**Ethical considerations**

All patients gave written, informed consent to ethically approved study protocols in the AVAST-M study; healthy volunteers were consented as part of study protocol ethics/12324 given favourable ethical opinion by the University of Manchester Senate Ethics Committee. Positive control patient tumor samples were collected with written full-informed patient consent under Manchester Cancer Research Centre (MCRC) Biobank ethics application #07/H1003/161+5 and approval for the work under MCRC Biobank Access Committee application 13_RIMA_01.

**Plasma collection**

Blood was collected in EDTA tubes as part of the AVAST-M study protocol, kept on ice and processed for plasma within 30 minutes of collection at 2000 rpm for 10 minutes at 4**°**C, then stored at -80**°**C until analysis.

**Droplet digital PCR**

CfDNA extracted from 30 healthy volunteer plasmas was used for assay validation and included as a negative control sample in each ddPCR assay. Assay specificity was tested in 60 reactions using healthy volunteer cfDNA and were all found to be negative.

Each negative control or patient sample was run over three replicate wells. Each well (22µl total) contained 8.8 µl of cfDNA sample, ddPCR Supermix for probes (no UTP) (Bio-Rad), wild-type and mutant probes (PrimePCR™ ddPCR™ Mutation Assay: BRAF WT for p.V600E, Human (dHsaCP2000028), PrimePCR™ ddPCR™ Mutation Assay: BRAF p.V600E, Human (dHsaCP2000027), PrimePCR™ ddPCR™ Mutation Assay: NRAS WT for p.Q61K, Human (dHsaCP2000068), PrimePCR™ ddPCR™ Mutation Assay: NRAS p.Q61K, Human (dHsaCP2000067), [PrimePCR™ ddPCR™ Mutation Assay: NRAS WT for p.Q61L, Human](http://www.bio-rad.com/en-uk/prime-pcr-assays/assay/dhsacp2000070-primepcr-ddpcr-mutation-assay-nras-wt-for-p-q61l-human) (dHsaCP2000070), [PrimePCR™ ddPCR™ Mutation Assay: NRAS p.Q61L, Human](http://www.bio-rad.com/en-uk/prime-pcr-assays/assay/dhsacp2000069-primepcr-ddpcr-mutation-assay-nras-p-q61l-human) (dHsaCP2000069) Bio-Rad) according to the manufacturer’s instructions. Droplets were generated using a QX200 Automated Droplet Generator (Bio-Rad) and a PCR reaction was performed using the following cycling conditions: 95°C for 10min; 40cycles of 94°C for 30sec and 55°C for 1min; followed by 98°C for 10min (all at a ramp rate of 2°C/sec), and a final hold at 4°C (ramp rate 1°C/sec). Droplets were read using a QX200 Droplet Reader (Bio-Rad) and the data analyzed using QuantaSoft version 1.4.0.99 software (Bio-Rad). All runs included negative controls of cfDNA extracted from 2ml of healthy volunteer plasma and positive control DNA extracted from cell lines or patient tumors with known mutational status (*BRAF* p.V600E from A375 [ATCC]; *NRAS* p.Q61K and *NRAS* p.Q61L from patient-tumors). Positive control samples were assayed in a single well using 2.2µl of input DNA (1ng/µl), ddPCR Supermix for probes (no UTP), appropriate wild-type and mutant probes and water.

**Statistical analyses**

Internal validation of ctDNA performed using bootstrapping with 1000 samples and the performance of the ctDNA model assessed using prognostic separation D statistics (PSDS) [1]. The performance of modelling the current AJCC prognostic variables (Breslow, ulceration, stage and N classification) compared to this model including ctDNA (i.e. adjusted for ctDNA) were assessed by determining discriminative and predictive ability through obtaining the PSDS, Nagelkerke R^2^ and assessing model calibration [1–3].

**Supplementary method references**

1. Royston P, Sauerbrei W. A new measure of prognostic separation in survival data. Stat. Med. 2004; 23(5):723–748.

2. Nagelkerke NJD. A Note on a General Definition of the Coefficient of Determination. Biometrika 1991; 78(3):691–692.

3. Van Houwelingen JC, Le Cessie S. Predictive value of statistical models. Stat. Med. 1990; 9(11):1303–1325.
