# Supplementary material for: Circulating tumor DNA predicts survival in patients with resected high-risk stage II/III melanoma
Source: Ann Oncol. 2017 Nov 3;29(2):490–6. doi: 10.1093/annonc/mdx717 (PMC5834029; doi:10.1093/annonc/mdx717)
Supplement: Supplementary Tables [file supplementary_tables_mdx717.docx]

**Supplementary tables**

**Supplementary Table 1 (S1).**

**Total number of copies and volume of plasma processed for individual patients.**

| **Patient**  **number** | **BRAF** | **NRAS** | **Base pair change** | **No. of ml plasma available** | **MT (total copies^a^)** | **WT (total copies^a^)** |
| --- | --- | --- | --- | --- | --- | --- |
| 11 | V600E | WT | 1799 T>A | 2 | 0 | 1964 |
| 14 | V600E | WT | 1799 T>A | 2 | 0 | 2186 |
| 27 | V600E | N/t | 1799 T>A | 2 | 0 | 2786 |
| 36 | V600E | N/t | 1799 T>A | 1.35 | 28.6 | 2766 |
| 40 | WT | Q>K | 181 C>A | 1.1 | 0 | 1258 |
| 44 | V600E | WT | 1799 T>A | 1.5 | 0 | 2746 |
| 58 | V600E | WT | 1799 T>A | 2 | 1.4 | 726 |
| 63 | WT | Q>K | 181 C>A | 2 | 0 | 2238 |
| 73 | V600E | WT | 1799 T>A | 2 | 0 | 2268 |
| 81 | V600E | N/t | 1799 T>A | 2 | 0 | 1758 |
| 87 | V600E | N/t | 1799 T>A | 1.5 | 0 | 1158 |
| 90 | V600E | N/t | 1799 T>A | 2 | 0 | 1812 |
| 101 | V600E | WT | 1799 T>A | 2 | 0 | 2132 |
| 114 | V600E | WT | 1799 T>A | 2 | 0 | 996 |
| 116 | V600E | WT | 1799 T>A | 2 | 1.4 | 2208 |
| 120 | WT | Q>L | 182 A>T | 1.9 | 0 | 8180 |
| 121 | V600E | N/t | 1799 T>A | 2 | 0 | 1760 |
| 133 | V600E | N/t | 1799 T>A | 1.7 | 0 | 1066 |
| 136 | V600E | N/t | 1799 T>A | 2 | 0 | 2280 |
| 137 | V600E | WT | 1799 T>A | 2 | 0 | 820 |
| 146 | V600E | N/t | 1799 T>A | 2 | 0 | 1012 |
| 158 | WT | Q>L | 182 A>T | 2 | 0 | 4024 |
| 160 | V600E | WT | 1799 T>A | 2 | 0 | 1370 |
| 168 | V600E | N/t | 1799 T>A | 2 | 0 | 2396 |
| 172 | V600E | WT | 1799 T>A | 1 | 0 | 2822 |
| 180 | V600E | N/t | 1799 T>A | 2 | 0 | 1550 |
| 182 | V600E | N/t | 1799 T>A | 2 | 0 | 3176 |
| 183 | V600E | N/t | 1799 T>A | 2 | 0 | 1952 |
| 198 | V600E | N/t | 1799 T>A | 1.9 | 298 | 29300 |
| 203 | V600E | WT | 1799 T>A | 2 | 0 | 7420 |
| 210 | V600E | N/t | 1799 T>A | 2 | 6.8 | 904 |
| 216 | V600E | N/t | 1799 T>A | 2 | 40.6 | 4022 |
| 223 | WT | Q>K | 181 C>A | 2 | 0 | 4550 |
| 229 | V600E | N/t | 1799 T>A | 1.7 | 1.6 | 2926 |
| 237 | WT | Q>K | 181 C>A | 1.3 | 4.4 | 1608 |
| 244 | WT | Q>K | 181 C>A | 2 | 1.4 | 1100 |
| 245 | WT | Q>K | 181 C>A | 2 | 0 | 1194 |
| 250 | V600E | N/t | 1799 T>A | 2 | 0 | 2608 |
| 263 | V600E | WT | 1799 T>A | 2 | 0 | 3330 |
| 264 | WT | Q>K | 181 C>A | 2 | 0 | 1456 |
| 274 | V600E | N/t | 1799 T>A | 2 | 0 | 3380 |
| 275 | V600E | WT | 1799 T>A | 2 | 0 | 1690 |
| 278 | V600E | N/t | 1799 T>A | 2 | 0 | 5006 |
| 279 | V600E | N/t | 1799 T>A | 2 | 0 | 7620 |
| 280 | V600E | N/t | 1799 T>A | 2 | 0 | 1518 |
| 287 | V600E | N/t | 1799 T>A | 2 | 0 | 1568 |
| 296 | WT | Q>K | 181 C>A | 2 | 0 | 1112 |
| 297 | V600E | WT | 1799 T>A | 2 | 0 | 1674 |
| 300 | V600E | N/t | 1799 T>A | 2 | 0 | 1280 |
| 308 | V600E | N/t | 1799 T>A | 1 | 0 | 796 |
| 311 | V600E | N/t | 1799 T>A | 1.6 | 0 | 1996 |
| 324 | V600E | WT | 1799 T>A | 2 | 0 | 4912 |
| 327 | V600E | WT | 1799 T>A | 1.75 | 0 | 9500 |
| 340 | V600E | N/t | 1799 T>A | 1.9 | 1.6 | 4430 |
| 341 | V600E | WT | 1799 T>A | 2 | 0 | 3176 |
| 342 | V600E | WT | 1799 T>A | 1.6 | 0 | 2050 |
| 347 | V600E | WT | 1799 T>A | 2 | 0 | 3482 |
| 355 | V600E | N/t | 1799 T>A | 2 | 0 | 2504 |
| 357 | WT | Q>K | 181 C>A | 2 | 0 | 1002 |
| 358 | WT | Q>K | 181 C>A | 1.2 | 3.4 | 710 |
| 365 | V600E | N/t | 1799 T>A | 2 | 0 | 1876 |
| 382 | V600E | WT | 1799 T>A | 2 | 0 | 1576 |
| 390 | V600E | N/t | 1799 T>A | 2 | 0 | 1744 |
| 392 | V600E | N/t | 1799 T>A | 2 | 0 | 3102 |
| 398 | V600E | N/t | 1799 T>A | 2 | 8 | 2314 |
| 402 | V600E | N/t | 1799 T>A | 2 | 0 | 2616 |
| 425 | V600E | WT | 1799 T>A | 1.25 | 0 | 7660 |
| 434 | V600E | N/t | 1799 T>A | 1 | 0 | 3018 |
| 443 | V600E | N/t | 1799 T>A | 2 | 0 | 1272 |
| 459 | V600E | N/t | 1799 T>A | 1.8 | 130 | 2288 |
| 467 | WT | Q>K | 181 C>A | 1.75 | 0 | 2300 |
| 469 | V600E | N/t | 1799 T>A | 2 | 0 | 1076 |
| 483 | V600E | N/t | 1799 T>A | 2 | 0 | 3732 |
| 484 | V600E | N/t | 1799 T>A | 1.9 | 0 | 1472 |
| 485 | V600E | N/t | 1799 T>A | 1.1 | 0 | 1738 |
| 487 | V600E | N/t | 1799 T>A | 1.4 | 0 | 4424 |
| 501 | V600E | N/t | 1799 T>A | 2 | 0 | 772 |
| 504 | V600E | N/t | 1799 T>A | 1.4 | 0 | 1048 |
| 514 | V600E | WT | 1799 T>A | 2 | 0 | 3062 |
| 526 | V600E | N/t | 1799 T>A | 2 | 0 | 38580 |
| 530 | V600E | N/t | 1799 T>A | 0.9 | 0 | 2272 |
| 532 | WT | Q>K | 181 C>A | 1.6 | 0 | 8860 |
| 536 | WT | Q>K | 181 C>A | 2 | 0 | 2958 |
| 540 | WT | Q>K | 181 C>A | 2 | 0 | 2320 |
| 543 | V600E | WT | 1799 T>A | 1.9 | 0 | 2560 |
| 550 | V600E | N/t | 1799 T>A | 2 | 0 | 346 |
| 556 | V600E | N/t | 1799 T>A | 0.9 | 0 | 1928 |
| 566 | V600E | N/t | 1799 T>A | 1.7 | 1.4 | 3128 |
| 568 | V600E | N/t | 1799 T>A | 2 | 2.8 | 2034 |
| 577 | V600E | WT | 1799 T>A | 2 | 0 | 2426 |
| 608 | V600E | N/t | 1799 T>A | 2 | 0 | 2410 |
| 611 | V600E | WT | 1799 T>A | 1.8 | 0 | 6360 |
| 612 | V600E | N/t | 1799 T>A | 2 | 0 | 2496 |
| 616 | V600E | N/t | 1799 T>A | 1.45 | 0 | 1652 |
| 638 | V600E | N/t | 1799 T>A | 2 | 0 | 2310 |
| 647 | V600E | N/t | 1799 T>A | 2 | 0 | 4202 |
| 650 | V600E | N/t | 1799 T>A | 2 | 0 | 2220 |
| 654 | WT | Q>L | 182 A>T | 2 | 0 | 858 |
| 659 | V600E | N/t | 1799 T>A | 2 | 0 | 5900 |
| 660 | V600E | N/t | 1799 T>A | 2 | 0 | 1860 |
| 688 | V600E | N/t | 1799 T>A | 2 | 0 | 578 |
| 691 | V600E | N/t | 1799 T>A | 2 | 0 | 426 |
| 696 | V600E | N/t | 1799 T>A | 1.25 | 0 | 934 |
| 705 | WT | Q>K | 181 C>A | 2 | 0 | 3560 |
| 715 | WT | Q>K | 181 C>A | 2 | 0 | 6480 |
| 719 | WT | Q>K | 181 C>A | 2 | 0 | 4402 |
| 728 | V600E | N/t | 1799 T>A | 1.9 | 0 | 4580 |
| 733 | V600E | N/t | 1799 T>A | 2 | 0 | 1960 |
| 737 | V600E | N/t | 1799 T>A | 1.6 | 0 | 984 |
| 745 | WT | Q>K | 181 C>A | 2 | 0 | 2100 |
| 765 | V600E | WT | 1799 T>A | 2 | 0 | 1784 |
| 767 | V600E | N/t | 1799 T>A | 1.3 | 0 | 806 |
| 771 | V600E | WT | 1799 T>A | 2 | 0 | 2008 |
| 783 | WT | Q>K | 181 C>A | 2 | 0 | 2432 |
| 788 | V600E | N/t | 1799 T>A | 2 | 0 | 3084 |
| 794 | V600E | WT | 1799 T>A | 2 | 0 | 7560 |
| 798 | WT | Q>K | 181 C>A | 2 | 0 | 2988 |
| 801 | V600E | N/t | 1799 T>A | 2 | 0 | 1366 |
| 836 | WT | Q>K | 181 C>A | 1 | 0 | 942 |
| 839 | V600E | N/t | 1799 T>A | 1.5 | 0 | 1122 |
| 857 | V600E | N/t | 1799 T>A | 2 | 0 | 2496 |
| 865 | V600E | WT | 1799 T>A | 2 | 1.4 | 3730 |
| 868 | V600E | WT | 1799 T>A | 2 | 0 | 1776 |
| 894 | V600E | N/t | 1799 T>A | 1.35 | 0 | 1444 |
| 917 | V600E | N/t | 1799 T>A | 1.7 | 0 | 3052 |
| 919 | V600E | N/t | 1799 T>A | 2 | 0 | 708 |
| 921 | V600E | N/t | 1799 T>A | 2 | 0 | 1980 |
| 933 | V600E | N/t | 1799 T>A | 1.075 | 0 | 448 |
| 959 | V600E | N/t | 1799 T>A | 2 | 0 | 906 |
| 965 | V600E | N/t | 1799 T>A | 1.9 | 0 | 2314 |
| 982 | V600E | N/t | 1799 T>A | 0.65 | 0 | 472 |
| 986 | V600E | N/t | 1799 T>A | 1.5 | 0 | 1294 |
| 1004 | WT | Q>K | 181 C>A | 1 | 0 | 572 |
| 1006 | V600E | N/t | 1799 T>A | 2 | 0 | 1398 |
| 1011 | V600E | N/t | 1799 T>A | 1.9 | 0 | 1838 |
| 1022 | WT | Q>K | 181 C>A | 1.4 | 0 | 1006 |
| 1025 | V600E | WT | 1799 T>A | 2 | 0 | 1392 |
| 1037 | V600E | N/t | 1799 T>A | 2 | 0 | 1744 |
| 1049 | V600E | N/t | 1799 T>A | 2 | 0 | 1770 |
| 1060 | V600E | N/t | 1799 T>A | 1.8 | 0 | 5600 |
| 1087 | V600E | N/t | 1799 T>A | 2 | 0 | 6700 |
| 1098 | V600E | N/t | 1799 T>A | 1.15 | 0 | 2446 |
| 1101 | V600E | WT | 1799 T>A | 2 | 1.4 | 3500 |
| 1125 | V600E | N/t | 1799 T>A | 1.75 | 0 | 964 |
| 1130 | V600E | WT | 1799 T>A | 1.85 | 0 | 5580 |
| 1136 | V600E | WT | 1799 T>A | 2 | 0 | 12520 |
| 1137 | WT | Q>K | 181 C>A | 2 | 0 | 1510 |
| 1146 | V600E | WT | 1799 T>A | 2 | 0 | 2818 |
| 1150 | V600E | N/t | 1799 T>A | 1.6 | 0 | 588 |
| 1154 | V600E | N/t | 1799 T>A | 2 | 0 | 1380 |
| 1163 | V600E | N/t | 1799 T>A | 2 | 0 | 7720 |
| 1164 | V600E | WT | 1799 T>A | 2 | 0 | 1320 |
| 1173 | WT | Q>K | 181 C>A | 2 | 0 | 3884 |
| 1176 | V600E | WT | 1799 T>A | 2 | 0 | 2308 |
| 1191 | V600E | WT | 1799 T>A | 2 | 0 | 1602 |
| 1194 | V600E | WT | 1799 T>A | 2 | 0 | 4466 |
| 1197 | V600E | N/t | 1799 T>A | 1.25 | 1.4 | 1234 |
| 1238 | V600E | WT | 1799 T>A | 2 | 0 | 4238 |
| 1242 | V600E | N/t | 1799 T>A | 1.65 | 0 | 1336 |
| 1243 | WT | Q>K | 181 C>A | 1.9 | 1608 | 4396 |
| 1326 | V600E | WT | 1799 T>A | 2 | 0 | 12340 |

^a^Poisson corrected according to manufacturers instructions (Bio-rad).

MT = mutant, WT = wild type N/t= not tested, ml = millilitre

**Supplementary Table 2 (S2).**

**Frequency of ctDNA copy numbers**

| **ctDNA copy number** | **N (%)** |
| --- | --- |
| **0** | 142 (88) |
| **1-3** | 10 (6) |
| **3.1-10** | 4 (2) |
| **10.1 -50** | 2 (1) |
| **50.1-1608** | 3 (2) |

**Supplementary Table 3 (S3).**

**Outcomes of patients with detectable and undetectable ctDNA**

|  | **Undetectable ctDNA** | | **Detectable ctDNA** | | **Total** |  |
| --- | --- | --- | --- | --- | --- | --- |
|  | **N** | **%** | **N** | **%** | **N** | **% of total** |
| **Total first local relapse** | 37 |  | 10 |  | 47 |  |
| **Site of first local relapse^a^** |  |  |  |  |  |  |
| Local recurrence at primary site | 14 | 38 | 2 | 20 | 16 | 34 |
| In transit metastases | 6 | 16 | 0 | 0 | 6 | 13 |
| Regional lymph node metastases | 12 | 32 | 5 | 50 | 17 | 36 |
| Multiple sites | 5 | 14 | 3 | 30 | 8 | 17 |
| **Subsequent therapy for first local relapse** |  |  |  |  |  |  |
| Immunotherapy | 1 | 3 | 0 | 0 | 1 | 2 |
| Targeted therapy | 3 | 8 | 0 | 0 | 3 | 6 |
| Chemotherapy | 0 | 0 | 1 | 10 | 1 | 2 |
| Radiotherapy | 6 | 16 | 1 | 10 | 7 | 15 |
| **Total first distant relapse** | 41 |  | 7 |  | 48 |  |
| **Site of first distant relapse^a^** |  |  |  |  |  |  |
| Soft tissue | 9 | 22 | 2 | 29 | 11 | 23 |
| Liver | 1 | 2 | 0 | 0 | 1 | 2 |
| Pulmonary | 6 | 15 | 0 | 0 | 6 | 13 |
| Brain | 3 | 7 | 1 | 14 | 4 | 8 |
| Bone | 1 | 2 | 0 | 0 | 1 | 2 |
| Other | 2 | 5 | 0 | 0 | 2 | 4 |
| Multiple sites | 19 | 47 | 4 | 57 | 23 | 48 |
| **Subsequent therapy for first distant relapse** |  |  |  |  |  |  |
| Immunotherapy | 2 | 5 | 0 |  | 2 | 4 |
| Targeted therapy | 9 | 22 | 0 |  | 9 | 19 |
| Chemotherapy | 8 | 20 | 3 |  | 11 | 23 |
| Radiotherapy | 10 | 24 | 3 |  | 13 | 27 |

^a^Patients can have simultaneous local/distant relapse.

**Supplementary Table 4 (S4).**

**Sites of relapse**

|  | **Undetectable ctDNA** | | **Detectable ctDNA** | | **Total** |  |
| --- | --- | --- | --- | --- | --- | --- |
|  | **N** | **%** | **N** | **%** | **N** | **% of total** |
| **Total first local relapse** | 37 |  | 10 |  | 47 |  |
| **Multiple sites of first local relapse** | 5 | 14 | 3 | 30 | 8 | 17 |
| Local recurrence at primary site and Regional lymph node metastases | 1 |  | 0 |  | 1 | 2 |
| Local recurrence at primary site, in transit metastases and Regional lymph node metastases | 2 |  | 2 |  | 4 | 9 |
| In transit metastases and regional lymph node metastases | 2 |  | 1 |  | 3 | 6 |
| **Total first distant relapse** | 41 |  | 7 |  | 48 |  |
| **Multiple sites of first distant relapse** | 19 | 47 | 4 | 57 | 23 | 48 |
| Soft tissue and Pulmonary | 5 | 12 | 0 | 0 | 5 | 12 |
| Soft tissue, liver and Pulmonary | 2 | 5 | 0 | 0 | 2 | 5 |
| Soft tissue, Pulmonary and brain | 1 | 2 | 0 | 0 | 1 | 2 |
| Soft tissue, Pulmonary, bone and brain | 1 | 2 | 0 | 0 | 1 | 2 |
| Soft tissue and bone | 1 | 2 | 0 | 0 | 1 | 2 |
| Soft tissue and other | 1 | 2 | 0 | 0 | 1 | 2 |
| Pulmonary and liver | 2 | 5 | 1 | 14 | 3 | 6 |
| Pulmonary and bone | 1 | 2 | 0 | 0 | 1 | 2 |
| Pulmonary and brain | 0 | 0 | 1 | 14 | 1 | 2 |
| Pulmonary, bone and other | 0 | 0 | 1 | 14 | 1 | 2 |
| Pulmonary and other | 2 | 5 | 0 | 0 | 2 | 4 |
| Liver and brain | 1 | 2 | 0 | 0 | 1 | 2 |
| Bone and other | 1 | 2 | 0 | 0 | 1 | 2 |
| Brain and other | 1 | 2 | 1 | 14 | 2 | 4 |
